# Molecular Evolution and Interaction of Membrane Transport and Photoreception in Plants

**DOI:** 10.3389/fgene.2019.00956

**Published:** 2019-10-11

**Authors:** Mohammad Babla, Shengguan Cai, Guang Chen, David T. Tissue, Christopher Ian Cazzonelli, Zhong-Hua Chen

**Affiliations:** ^1^School of Science and Health, Western Sydney University, Penrith, NSW, Australia; ^2^College of Agriculture and Biotechnology, Zhejiang University, Hangzhou, China; ^3^Hawkesbury Institute for the Environment, Western Sydney University, Penrith, NSW, Australia

**Keywords:** light, photoreceptors, membrane transporters, membrane potential, ion flux, crop nutrition

## Abstract

Light is a vital regulator that controls physiological and cellular responses to regulate plant growth, development, yield, and quality. Light is the driving force for electron and ion transport in the thylakoid membrane and other membranes of plant cells. In different plant species and cell types, light activates photoreceptors, thereby modulating plasma membrane transport. Plants maximize their growth and photosynthesis by facilitating the coordinated regulation of ion channels, pumps, and co-transporters across membranes to fine-tune nutrient uptake. The signal-transducing functions associated with membrane transporters, pumps, and channels impart a complex array of mechanisms to regulate plant responses to light. The identification of light responsive membrane transport components and understanding of their potential interaction with photoreceptors will elucidate how light-activated signaling pathways optimize plant growth, production, and nutrition to the prevailing environmental changes. This review summarizes the mechanisms underlying the physiological and molecular regulations of light-induced membrane transport and their potential interaction with photoreceptors in a plant evolutionary and nutrition context. It will shed new light on plant ecological conservation as well as agricultural production and crop quality, bringing potential nutrition and health benefits to humans and animals.

## Introduction

Light is one of the most important environmental cues for plant growth and development and other physiological responses *via* the changes of intensity and spectral quality of light (Steinger et al., 2003; Fan et al., 2013). In natural environments, plants regularly experience rapid changes in the intensity of solar radiation during the day and across seasonal changes. Plant leaves have evolved morphological adaptations and physiological acclimation processes to survive light levels ranging from nearly 0 (i.e., darkness) up to 2,000 µmol m^−2^ s^−1^ photons during the peak of midday irradiances. High irradiances generally reduce specific leaf area, increase leaf thickness, alter palisade cell development, and change the position and composition of chloroplasts (Karpinski et al., 1999; Givnish et al., 2004; Walters, 2005; Matos et al., 2009). Light is used as photosynthetic energy source as well as a vital regulator that can affect plant photomorphogenesis through the perception of the spectrum regulated by photoreceptors and potentially the downstream signaling component-membrane transporters. Membrane transport plays a central role virtually in all the aspects of plant ion and solute homeostasis and signaling transduction (Wang et al., 2018). Specialized plant membrane transporters are a promising target to increase crop yields, enhance produce quality, and improve resistance to abiotic and biotic stresses for the sustainable production of nutritious foods (Schroeder et al., 2013). However, the underlying light sensory mechanisms of plants are complex. In this review, we summarize how light regulates plant functions at the molecular, cellular, tissue, and whole-plant levels. We highlight how photoreceptors may have profound regulation on membrane transport in plant cells. We emphasize how coordinated light responses drive ion exchange, membrane transporters, and photoreceptors to promote leaf and fruit developments for improving crop nutrition.

### Molecular Physiology of Light Response in Plants and Fruits

Light is used in the photosynthetic machinery, as well as in various regulatory processes such as seed germination, flowering, stomatal development, and membrane transport of guard cells (Assmann et al., 1985; Talbott and Zeiger, 1993; Walters et al., 2003; Fan et al., 2004; Lee et al., 2007b). The responses of plants to light at the whole plant, organ, cell, and molecular levels are evolutionarily conserved (Mullineaux and Karpinski, 2002; Christie, 2007; Shimazaki et al., 2007). Light is heterogeneously distributed throughout the canopy in a horizontal and the vertical planes, but a more homogenous light distribution in the canopy can be advantageous (Li et al., 2014). Moreover, excess light is a stress that can damage DNA and other cellular components, leading to the detrimental effect on photosynthesis (Takahashi and Badger, 2011; Jenkins, 2017; Yin and Ulm, 2017). Plants have mechanisms to protect against excess light that triggers photo-oxidative and harmful damage by minimizing the exposure of photosynthetic tissue to excessive radiation (Martín et al., 2016) and therefore acclimate to the different light wavelengths, showing a high degree of morphological and physiological plasticity (O’carrigan et al., 2014). For example, plants have adapted to excessive light through curling their leaves (Neuner et al., 1999), enhancing their cuticular wax, and altering leaf as well as whole-plant morphology (Horton et al., 1996). Plants can also reduce their specific leaf area (SLA) when photosynthetic photon flux density is increased from 50 to 500 µmol m^−2^ s^−1^ (Fan et al., 2013). These alterations in leaf morphology can essentially regulate plant growth, photosynthesis, fruit development, yield, and quality (Azari et al., 2010).

Light has a very important role in determining the nutrient level of plant-based foods, thus affecting human nutrition and health (Davis and Uthus, 2004; Evans et al., 2006). For instance, light treatments can significantly regulate the accumulation of micronutrients and pigments in tomato (O’carrigan et al., 2014). Plant pigments (chlorophyll and carotenoids) are light-sensitive and intrinsic to absorption within photosystems I and II (PSI and PSII) (Gross, 2012). The illumination with high blue light increases the synthesis and accumulation of anthocyanins and carotenoids in pepper plants (*Capsicum annuum* L.) without affecting the flavonoid contents (Hoffmann et al., 2016). Fruit dry matter and soluble sugar content and the production of lycopene are improved by increasing solar irradiances which attribute to improved tomato fruit quality (Davies et al., 1981), but the excessive light can inhibit the lycopene content (Brandt et al., 2006). Improved amounts of ascorbate, lycopene, carotene, rutin, caffeic acid, and soluble phenol derivatives in fruit have been reported under increased light irradiance (Wilkens et al., 1996; Gautier et al., 2008). Moreover, the accumulation of anthocyanin and soluble sugar in strawberry fruit was promoted with the increases in the expression of aroma-related genes in light accompanied by the regulation of *FvMYB10* at transcriptional and post-translation levels (Xu et al., 2018). Naturally occurring plant derivative antioxidants, such as phenols, vitamins, carotenoids, and terpenoids, are likely to be beneficial significantly for health promotion with the reduction of inflammation associated with chronic diseases (Goggs et al., 2005). Plant extracts and their purified compounds are selectively turning off inflammatory *cyclooxygenase-2* (*COX-2*), while preserving housekeeping gene *cyclooxygenase-1* (*COX-1*) (Evans et al., 2006). Thus, nutrient-rich foods are likely to be regulated by light through plant photoreceptors and membrane transport of nutrient to the eatable parts of the plants, which are very multifaceted processes worth investigating in the future.

Plant response to light is modulated by large number of light-regulated genes, which encode proteins including photoreceptors, early signaling components, pleiotropic constitutive photomorphogenic/de-etiolated/fusca (COP/DET/FUS), and many downstream effectors (Quail, 2002; Jiao et al., 2007; Azari et al., 2010). Abrupt changes in gene expression due to excess light have been reported in plants (Rossel et al., 2002; Kimura et al., 2003; Murchie et al., 2005; Adamiec et al., 2008) and green algae (Im et al., 2003; Fischer et al., 2006). Gene expression is severely damaged when light intensities are greater than the maximum potential of the chloroplast electron capacity (Demarsy et al., 2018; Foyer, 2018). Light can enhance the repositioning of the *Arabidopsis* light-inducible *chlorophyll a/b-binding* (*CAB*) locus quickly from the nuclear interior to the nuclear periphery through the red/far-red *phytochromes* (*PHYs*) during its transcriptional activation (Feng et al., 2014). Likewise, the light-inducible *ribulose-1,5-bisphosphate carboxylase/oxygenase small subunit* (*RBCS*), *plastocyanin* (*PC*), and *genomes uncoupled 5* (*GUN5*) showed similar repositioning behavior upon their activation (Feng et al., 2014). Light-intensity-dependent changes of *light-harvesting chlorophyll protein complex associated with photosystem II* (*LHCIIs*) are regulated by *CAB* gene transcription (Escoubas et al., 1995). Moreover, regulation of microRNAs in light stress responses and adaptive mechanisms in plants have been emphasized and may be a potential target for light stress tolerance in crop plants (Yang et al., 2019). Integrative omics strategies, including both proteome and transcriptome, showed that different irradiances coordinate light harvesting, electron transport, and protein synthesis to cope with ever-changing environmental conditions by adjusting the thylakoid membrane proteome in pea (Albanese et al., 2018).

Photoreceptors especially phototropins (PHOTs) can modulate the expression of a large number of photosynthesis-related genes, such as *light-harvesting chlorophyll a/b-binding 1* (*LHCB1*), *ribulose-1,5-bisphosphate carboxylase/oxygenase small subunit* (*RBCS)*, *photosystem II manganese-stabilizing protein* (*PSBO*), *photosystem I reaction center subunit IV* (*PSAE*), and *photosynthetic electron transfer C* (*PETC*) (López-Juez et al., 2007). Plants grown at high light show up-regulation of *RBCS* and down-regulation of *LHCB1* in *Arabidopsis phot1-1* (*nph1*-1) and *phot2-5* (*npl1-1* or *cav1-5*) double mutants (López-Juez et al., 2007). In *Arabidopsis*, a subset of genes including *early light-inducible protein1* (*ELIP1*) and *ELIP2*, which encode light stress-related relatives of the *LHC* protein family and *production of anthocyanin pigments 1 and 2* (*PAP1*) and *PAP2*, seems to be regulated in excess light or high-intensity blue light by *cry1* (*cryptochrome 1*) mutant (Kleine et al., 2007). In addition, excess light-dependent regulation of 77 genes is changed in the *cry1* mutant, and 26 of these genes are also mis-regulated in an elongated *hypocotyl5* (*hy5*) transcription factor mutant (Kleine et al., 2007). The zinc-finger transcription factor *ZAT12/RHL41* can be rapidly induced in *Arabidopsis* to adapt in high light environment (Iida et al., 2000). Notably, blue light modulates the accumulation of anthocyanin in *Arabidopsis* seedlings, which is CRY-dependent (El-Esawi et al., 2017), and the overexpression of CRY1a increased the accumulation of anthocyanin in tomato (Liu et al., 2018).

In *Arabidopsis* about 10% (∼2,500) of the genes are modulated by PHYs under long-term light exposure (Tepperman et al., 2006). The PHY-regulated transcription of photoresponsive genes leads to photomorphogenesis *via* negative transcriptional factors such as PHY-interacting factors (PIFs) and positive transcriptional factors such as HY5 (elongated hypocotyl 5) and HYL (HY5-like) (Lee et al., 2007a; Leivar and Monte, 2014). Nonetheless, recent advances in light-induced modulation of photoreceptors have provided ample knowledge of their molecular interaction with various photosynthesis-related proteins in light signal transduction pathway.

Little is known about the direct link between photoreceptors and membrane transport along the signaling cascade, but circadian clocks appear to be involved in linking photoreceptors and solute transport (Haydon et al., 2011). Light input to the circadian clock and oscillator is mediated by the cryptochromes (CRYs) and PHYs (Somers et al., 1998; Devlin and Kay, 2000). It was reported that blue light and CRY-dependent circadian regulation of the sigma transcription factor (SIG5) form a part of the chloroplast signaling pathway in light stress adaptation (Belbin et al., 2017). In the transcription translation loops, light increases the promoter activity of *circadian clock-associated 1* (*CCA1*) and *late elongated hypocotyl* (*LHY*), while blue light directly activates Zeitlupe (ZTL) to bind GIGANTEA (GI). Interaction with GI stabilizes ZTL and ZTL and GI disassociates allows ZTL to target timing of CAB expression 1 (TOC1) for degradation at dusk (Dodd et al., 2007; Kim et al., 2007; Haydon et al., 2011). Therefore, light activates photoreceptors, and circadian regulation of transcripts may further regulate membrane transporters mediating fluxes of ions, sugars, and metabolites for plant nutrition.

#### Ion Fluxes in Response to Light

Understanding the physiological implications of light-induced electrical signaling at cellular and tissue levels is essential to elucidate the ionic balance and mineral nutrients at whole-plant level. Light can trigger a cascade of electrical events in thylakoid and plasma membranes of green plant tissues (Vredenberg and Tonk, 1975; Hansen et al., 1987; Elzenga et al., 1995; Johannes et al., 1997). At the plasma membrane, light induces voltage changes in *Arabidopsis* and ion fluxes of tobacco mesophyll protoplasts (Spalding et al., 1992; Blom-Zandstra et al., 1997). The kinetics of H^+^, Ca^2+^, K^+^, and Cl^−^ fluxes of the mesophyll and epidermis of broad bean is related to light-induced changes in the plasma membrane potential (E_m_) (Shabala and Newman, 1999). Light-activated depolarization in the mesophyll and epidermis is very different (Elzenga et al., 1995; Shabala and Newman, 1999). PHYs can alter the permeability of the plasma membrane to ions from the subsequent changes in E_m_ in response to light (Newman and Briggs, 1972; Racusen and Galston, 1983). However, the mechanisms underlying the light-induced transient E_m_ changes and their ionic basis remain elusive due to the limited number of studies.

Fruit quality that contributes to nutritional attributes can be modulated through light-induced ion fluxes with the coordinated interaction of membrane transporters. Light spectral composition is important factors in determining grape juice acidity and K^+^ concentrations (Shabala and Wilson, 2001), and fruit pH and K^+^ levels are modulated by leaf, instead of fruit, exposure to light (Morrison and Noble, 1990). Fluctuations in light intensity can considerably regulate net ion fluxes from and into the berry mesocarp, including changes in fruit apoplastic pH and K^+^ concentrations, which also play important role in chlorophyll-mediated light transduction to modulate light-dependent ionic exchange (Shabala and Wilson, 2001). It is proposed that K^+^ enters the cells in exchange for protons derived from organic acids, driven by light-induced membrane-bound H^+^-ATPase activity (Boulton, 1980a), which results in juice with lower acidity (Boulton, 1980b). However, further studies are required to identify and characterize membrane transporters in fruit tissue that are very different from leaf and root tissues of plants.

#### Proton Pumps and Light Response in Plants

**Figure 1:**
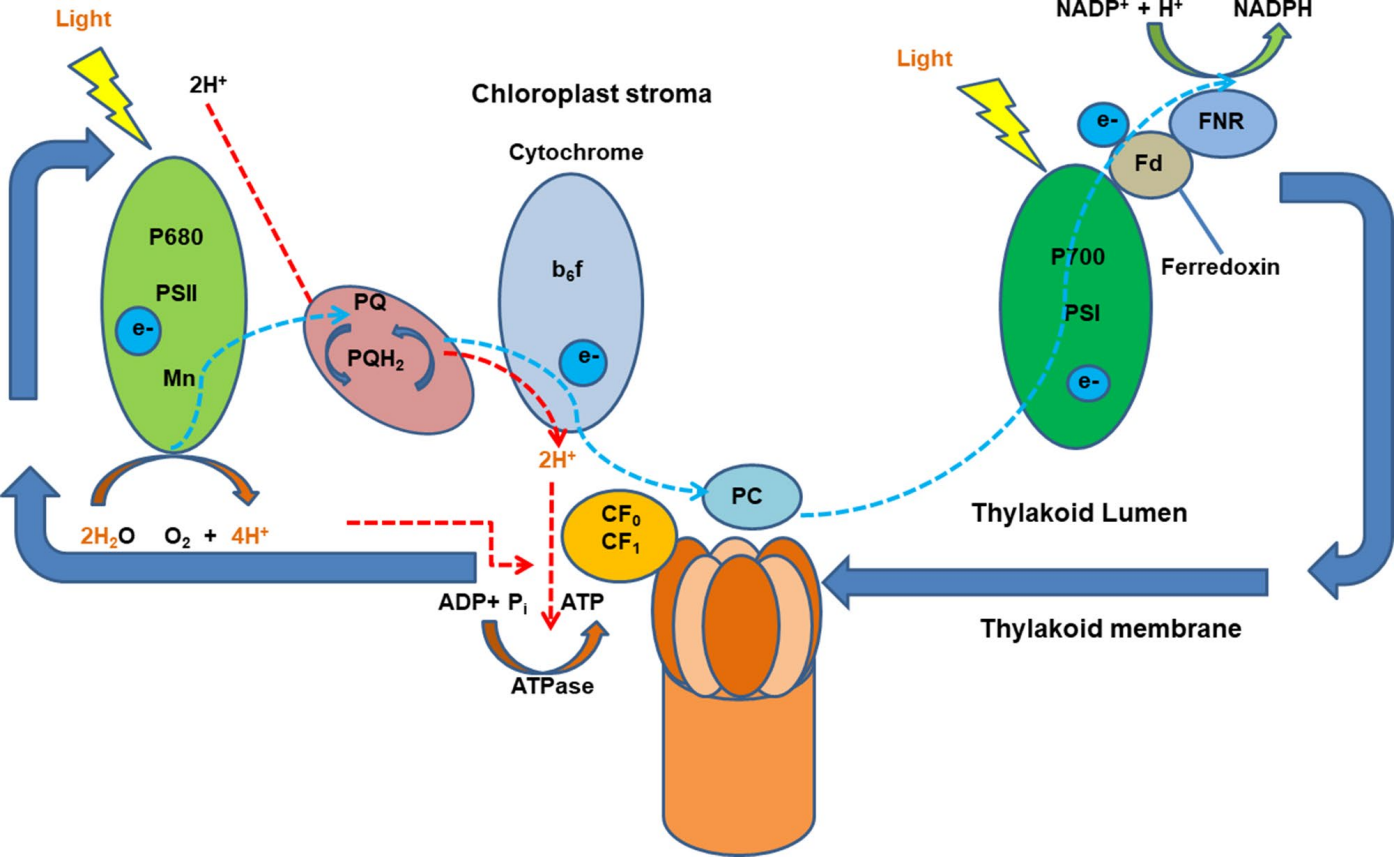
A schematic diagram of light-induced generation of proton motive force in chloroplasts. Light-generated electrons, transferred upon illumination, activate H^+^ pumping into the thylakoid lumen the electrochemical potential gradient which is driven by proton motive force (PMF) across membranes. Ion channels and transporters can then be regulated by PMF. Adapted and modified from (Muñiz et al., 1995). ADP, adenosine diphosphate; ATP, adenosine triphosphate; ATPase, ATP synthase; b6f, cytochrome b6f complex (plastoquinol–plastocyanin reductase); FNR, feradoxin NADPH reductase; Fd, ferredoxin; NADP/NADPH, nicotinamide adenine dinucleotide phosphate; P700, photosystem I primary donor; PSI, photosystem I; PSII, photosystem II.

In plants, proton gradients produced by primary H^+^-translocating pumps that hydrolyze either ATP [plasma membrane P-type H^+^ translocating ATPase (H^+^-ATPase) and tonoplast V-type H^+^ translocating ATPase (V-ATPase)] or PPi [tonoplast H^+^ translocating pyrophosphatase (V-PPase)] as the energy source to extrude protons, generating a proton motive force (PMF) to energize the membrane transport (Drozdowicz and Rea, 2001; Palmgren, 2001; Sze et al., 2002; Cheng et al., 2003). Numerous studies have shown an increase in the activity of the H^+^ pumping out of the cytosol to the apoplast in plants after the onset of illumination. Large light-induced H^+^ extrusion promotes leaf expansion by increasing cell growth and wall extensibility and regulates photosynthesis and ATP synthesis (Gay and Hurd, 1975; Walters et al., 2003; Christie, 2007; Kangasjärvi et al., 2012). Briefly, the light-induced electrogenic reactions take place in photosynthetic reaction centers of photosystems I and II (PSI and PSII). The light-induced electron transfer in PSII releases H^+^ into the thylakoid lumen and reduces plastoquinone molecule (PQ), which takes H^+^ from the outside and delivers them as PQH_2_ to the inner side of the membrane. Light-driven electrons activate H^+^ pumping from the stroma into the thylakoid lumen against the electrochemical potential which is driven by PMF across membranes. Protons translocate ATPase for ATP synthesis in response to the pH gradient is then established across the thylakoid membrane (Junge and Jackson, 1982; Muñiz et al., 1995) (**Figure 1**). It was reported that blue light–stimulated stomatal opening in the leaf is correlated with activation of an electrogenic pump in the guard cell which results in hyperpolarization of 45 mV and outward H^+^ currents of 5.5 pA, creating an electrochemical gradient for passive ion fluxes (Assmann et al., 1985). Red light also stimulates an outward current mediated by an electrogenic proton pump of *Vicia* guard cells *via* the modulation of chloroplasts (Serrano et al., 1988). However, whether there are any interactions between the blue and red light–induced proton pumping and photoreceptors are still not fully explored.

#### Ion Channels and Light Response in Plants

The movement of the ionic substrates is facilitated across the lipid bilayer by the ion channels (Spalding, 2000). Ion channels take part in sensory perception, ion homeostasis, secretion, and E_m_ regulation in all plant cells and play a vital role in the physiology of cells and whole organisms (Assmann et al., 1985; Serrano et al., 1988; Chen et al., 2012b; Hills et al., 2012). An early response of plant cells to blue and red light also results in the hyperpolarization of E_m_ and high proton pump activities (Assmann et al., 1985; Serrano et al., 1988), which affect ion channels. Here, we summarize ion channels that are important for light response in plant cells.

Potassium channels are an essential element controlling plant growth and development and affecting the homeostasis of other elements. In *Arabidopsis*, plasma membrane inward K^+^ channels (e.g., KAT1, KAT2, AKT1, AKT2) and outward K^+^ channels (e.g., GORK, SKOR) mediate K^+^ fluxes (Blom-Zandstra et al., 1997; Pilot et al., 2003; Zhao et al., 2018a). White light induces an increase in plasma membrane K^+^-channel activity and a 30- to 70-mV transient membrane depolarization (completed in 2–3 min) in *Arabidopsis* leaf mesophyll cells (Spalding et al., 1992). *Arabidopsis* two-pore K^+^ channels (TPKs) are localized to the tonoplast (TPK1, TPK2, TPK3, and TPK5) and plasma membrane (TPK4) (Voelker et al., 2006; Pfeil et al., 2014). *Arabidopsis tpk3* mutant has reduced generation of PMF, which results in altered thylakoid membrane organization, less CO_2_ assimilation, and reduced dissipation of excess light. Therefore, TPK3 modulates the composition of the PMF, necessary to convert photochemical energy into physiological functions, through ion counterbalancing (Carraretto et al., 2013). Moreover, the links between blue light receptor CRYs and their electrical responses in facilitating K^+^ channels involved in the blue light perception mechanism are reported (Suh et al., 2000), and red light–induced E_m_ transients in the moss *Physcomitrella patens* promote K^+^-channel interaction with PHY signaling (Johannes et al., 1997).

Calcium channels play key role in maintaining cellular response to different environmental stimuli perception. Depolarization-activated Ca^2+^ channels and hyperpolarization-activated calcium channels have been described and characterized in plants (Véry and Davies, 2000; Miedema et al., 2001), providing a pathway for Ca^2+^ influx into plant cells (Fairley-Grenot and Assmann, 1992; Marshall et al., 1994; White, 1994; Gelli and Blumwald, 1997). Uptake of Ca^2+^ in plant cells is accomplished through the depolarization and hyperpolarization-activated calcium channels orchestrated with non-selective cation channel, as well as Ca^2+^-ATPases and cotransporters acting in concert (Miedema et al., 2001). Early cellular responses to blue and red lights are attributed to the activation of Ca^2+^ permeable channels to mediate the influx of Ca^2+^ into cells such as guard cells (Hamilton et al., 2000) and root hair cells (Véry and Davies, 2000). However, full molecular characterization of these Ca^2+^ channels has yet to be explored in plants. Therefore, the regulations of light and photoreceptors on these Ca^2+^ channels are most likely to be indirect *via* the light regulation of PM H^+^-ATPase and potentially Ca^2+^-ATPase and ion homeostasis. Their links with light and photoreceptors are a challenge worthy of further discovery.

Plants have at least three families of genes encoding anion channels: slow anion channels (SLACs/SLAHs), rapid anion channels (ALMTs/QUACs), and chloride channels (CLCs) (Spalding, 2000; Roberts, 2006). Both SLACs and ALMTs can be activated by cytosolic Ca^2+^ and are permeable to a range of anions, including Cl^−^, malate^2−^, and NO^3−^ (Chen et al., 2007; Marten et al., 2007). Blue light not red light activates anion channels residing at the plasma membrane of hypocotyl cells of etiolated *Arabidopsis* seedlings. Anion channel–blocker 15-nitro-2-(3-phenylpropylamino)-benzoic acid (NPPB) inhibits the anion channels and reduces the blue-light-induced depolarization (Cho and Spalding, 1996). Electron transferred to receiver molecule from an excited CRY1 flavoprotein in redox reactions indicate that anion channels may be directly modulated by blue light (Cashmore et al., 1999). The early and important signal transduction role of the anion channels in the blue-light responses of seedling stems are also supported by the findings in *Arabidopsis* (Noh and Spalding, 1998), pea (Elzenga et al., 1995), and bean (Shabala and Newman, 1999). Illumination triggers a Ca^2+^-dependent anion channel in the plasma membrane of emerging pea leaf mesophyll cells (Elzenga and Van Volkenburgh, 1997; Lewis et al., 1997). Moreover, an *Arabidopsis* thylakoid membrane-localized voltage-dependent Cl^−^ channel (VCCN1) fine-tunes PMF *via* anion influx into the lumen in response to sudden changes to high light, implicating its involvement in photo-protective mechanisms (Herdean et al., 2016). In addition, the independent roles of ion transporters such as Cl^−^ channel e (CLCe), voltage-dependent Cl^−^ channel (VCCN1), and the K^+^/H^+^ antiporter (KEA3) have been unraveled in *Arabidopsis* to fine-tune photosynthesis in the fluctuating light environments (Dukic et al., 2019). However, it is not clear whether any other light receptors are involved in this process.

#### Co-Transporters and Light Response in Plants

For co-transporters, plants mostly use protons as the coupling ion to transport cations and anions across biological membranes driven by H^+^ gradient created by H^+^-ATPase, V-ATPase, and V-PPase (Sze et al., 1999; Gaxiola et al., 2002). For instance, *Arabidopsis* Ca^2+^/H^+^ exchanger 1 (CAX1) mutant *cax1* reduces tonoplast Ca^2+^/H^+^ antiporter activity about 50%, tonoplast V-ATPase activity 40%, and tonoplast Ca^2+^-ATPase activity 36% (Cheng et al., 2003). Plants perceive light absorbing far-red (FR) and red light (R) *via* the PHYA-E. The disrupted gene in *long after FR* (*laf6*) mutant encodes ATP-binding-cassette 1 (AtABC1) co-transporter that contains an N-terminal transit peptide targeting to chloroplasts. Mutation in *AtABC1* results in an accumulation of protoporphyrin IX and in attenuation of FR-regulated gene expression, which may act as a light-specific signal coordinating communication between plastids and the nucleus (Møller et al., 2001).

Membrane-localized sugar transporters (SUTs, STPs, or SUCs) facilitate the transport, storage, and utilization of sugars for plant growth and development (Gahrtz et al., 1994; Riesmeier et al., 1994; Srivastava et al., 2008). Expression of the AtSTP1 in guard cells showed a strong increase of AtSTP1 expression in the dark and a transient, diurnally regulated increase during the photoperiod around midday (Stadler et al., 2003; Weise et al., 2008). In *Solanum tuberosum*, StSUT1, StSUT2, and StSUT4 are co-localized, and their RNA levels not only follow a diurnal rhythm but also oscillate in constant light. The phenotype of *StSUT4-RNAi* plants includes early flowering, higher tuber production, and reduced sensitivity toward light enriched in far-red wavelength, indicating an indirect interaction of PHYs (or potential CRYs, PHOTs) and StSUS4 *via* the circadian clock genes (Chincinska et al., 2008).

Moreover, co-transporters across the chloroplast thylakoid membrane also play an essential role in light regulated cellular response and plant growth. *Arabidopsis* K^+^ efflux antiporter (KEA3) is critical for high photosynthetic efficiency on a shift from dark to low light (or high to low light) and *kea3* mutants show prolonged dissipation of absorbed light energy as heat (Armbruster et al., 2014; Kunz et al., 2014). Besides, *Arabidopsis* K^+^ efflux antiporters (KEAs) localized to the Golgi apparatus play a pivotal role in the maintenance of ionic and pH homeostasis and in coping with high K^+^/Na^+^ stress during quick etiolated growth of seedlings (Wang et al., 2019).

The thylakoid ATP/ADP carrier (TAAC) supply ATP for the nucleotide-dependent reactions in the thylakoid lumen, which facilitate the repair of PSII when plants are under light stress (Yin et al., 2010; Spetea and Lundin, 2012). To develop a comprehensive light signaling network, future investigations are required to discover new transporters responding and/or involved in light-mediated signaling and their interactions with photoreceptors.

### Light Response of Photoreceptors

**Figure 2:**
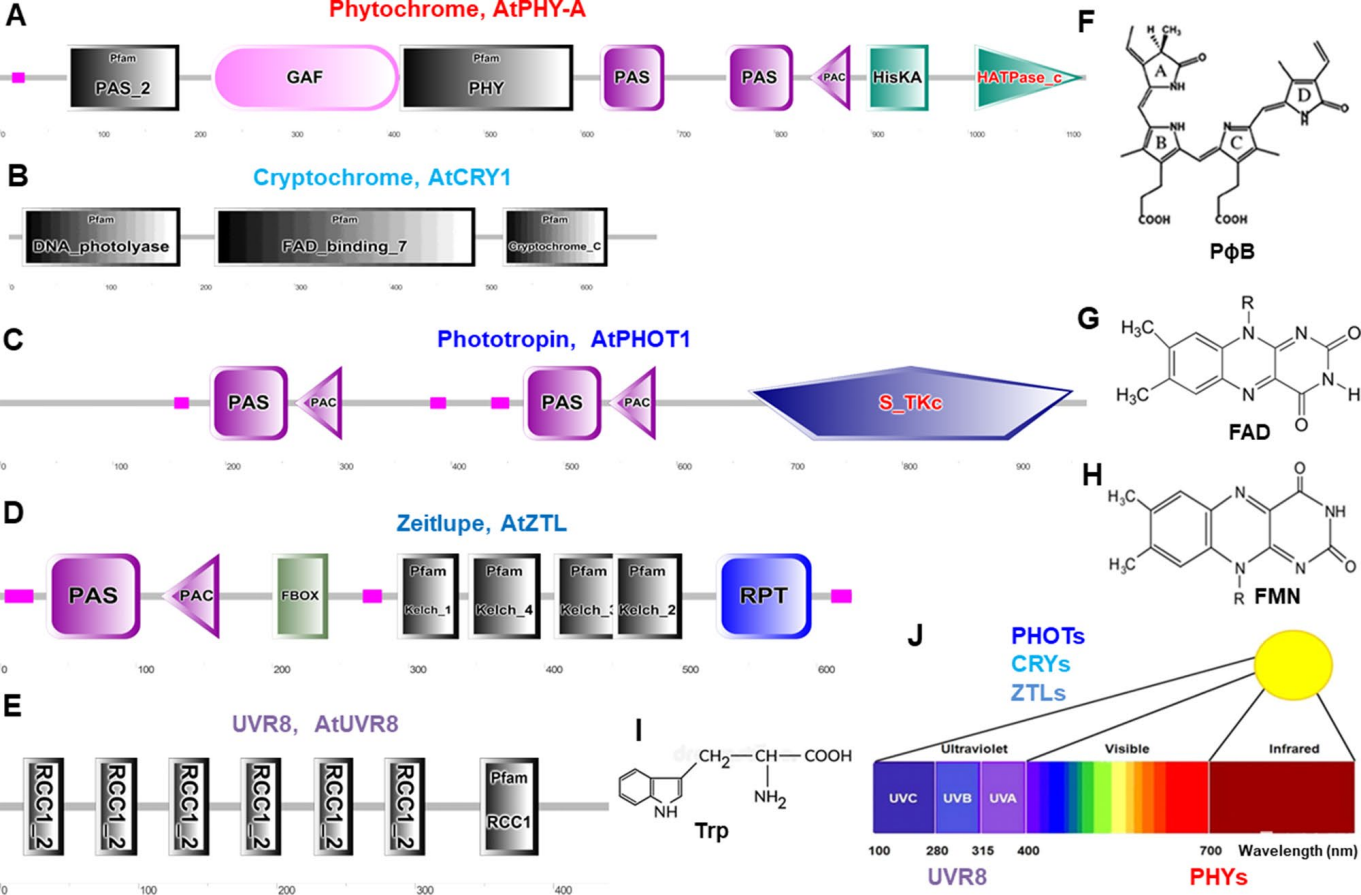
Schematic diagram of domain organization and structure of typical photoreceptors in *Arabidopsis*. These protein domains are for phytochromes **(A)**, cryptochromes **(B)**, phototropins **(C)**, Zeitlupes **(D)**, and UVR8 **(E)**. Molecular structures are phytochromobilin (PɸB, **F**), flavin adenine dinucleotide (FAD, **G**), flavin mononucleotide (FMN, **H**), and tryptophan (Trp, **I**). **(J)**, Light spectrum and the photoreceptors. Protein domain mages are generated from http://smart.embl–heidelberg.de/ and molecular structures are from (Galvão and Fankhauser, 2015). PAS, Per-Arndt-Sim; GAF, cGMP phosphodiesterase/adenyl cyclase/Fh1A; PAC, photoactivatable adenylyl cyclase; HisKA, histidine phosphorylation site in the histidine kinase domain; S_TKc, serine/threonine protein kinases protein kinases; F-box, F-box like domain; Kelch, kelch motif; RPT, root phototropism; FAD, flavin-adenine-di-nucleotide, UVR8, UV resistance locus 8; RCC1, regulator of chromosome condensation repeat1.

Light signals are sensed and utilized by a few major families of photoreceptors to initiate plant physiological responses through signal networks in integration with other environmental cues (Franklin et al., 2004). In plants, most photoreceptor families (**Figure 2**) consist of more than one member and share a high degree of resemblance among the different members (Kharshiing and Sinha, 2015). Furthermore, photoreceptors consist of photoreceptive domains binding chromophores to absorb light signals (**Figure 2**). Thus, the physical light signal is converted to a biochemical signal such as protein–protein interactions and enzyme activation by the photoreceptors to propagate downstream signal transduction (Kong and Okajima, 2016). Photoreceptors play a vital role in plants, regulating most of the spectral responses coupled with altering the expression of up to 1/3 of genes and proteins (Ma et al., 2001; Tepperman et al., 2001; Folta et al., 2003; Tepperman et al., 2004; Wang and Folta, 2013). Light induces extensive reprogramming of gene expression patterns in plants with the association of photoreceptors (Mawphlang and Kharshiing, 2017).

Phytochromes absorb wavelengths of red (600–700 nm) and far-red light (700–750 nm) and can optimize plant growth and development in changing light (Li et al., 2011; Xu et al., 2015; Hoang et al., 2019) and regulate germination, de-etiolation, shade avoidance responses, and many other plant physiological responses (Kong and Okajima, 2016). PHYs can be homodimeric or heterodimeric proteins comprising of a N-terminal photosensory region covalently bound to a phytochromobilin tetrapyrrole chromophore (PɸB), and a C-terminal output region involved in dimerization (**Figures 2A, F**) (Burgie and Vierstra, 2014). PHYs are encoded by the *PHYA-E* gene family in *Arabidopsis* and other plant species (Sharrock and Quail, 1989; Alba et al., 2000; Staiger, 2008) and are localized in the cytosol in dark to be imported into the nucleus upon activation by light (Nagatani, 2004). Most PHY responses are likely to be the interaction of activated PHYs with transcription factors within the nucleus and triggering responses in the cytosol (Kevei et al., 2007; Rösler et al., 2007; Fankhauser and Chen, 2008).

CRYs absorb wavelengths of blue light/ultraviolet (UV)-A (315–500 nm) and become increasingly evident as key regulators for plant stress responses under environmental fluctuation (Mishra and Khurana, 2017; Carvalho and Damião, 2018), such as inhibition of hypocotyl elongation, photoperiodic control of floral initiation, and circadian rhythms (Christie et al., 2014). CRYs relay light input sensed by a flavin adenine dinucleotide (FAD) chromophore to regulate different biological processes (**Figures 2B, G**) (Chaves et al., 2011). In *Arabidopsis*, UV-A and blue light–activated CRY1, CRY2, (Ahmad and Cashmore, 1993; Lin, 1996) CRY3, (Kleine et al., 2003; Staiger, 2008), and CRY-DASH (*Drosophila*, *Arabidopsis*, *Synechocystis*, and human) (Eder and Cosio, 1994) are well characterized. CRYs show different expression pattern and protein stability (Ahmad et al., 1998), and interaction of CRY1 with the E3-ubiquitin ligase constitutive photomorphogenic1 (COP1) is a major event to trigger UV-A and blue light–dependent changes in gene expression (Jiao et al., 2007).

Phototropins, plasma membrane-associated kinases, are activated by blue light/UV-A (315–500 nm) (Christie, 2007; Hart et al., 2019) and play important physiological roles in optimizing plant photosynthetic capacity *via* mediating differential cell growth in the phototropic response, chloroplast movement, stomatal opening, and leaf flattening (Matsuoka and Tokutomi, 2005; Inoue et al., 2008; Christie et al., 2014). PHOTs’ primary structure consists of an N-terminal photosensory region and a C-terminal AGC type Ser/Thr protein kinase domain. Two flavin mononucleotide (FMN) chromophore bound to light oxygen voltage (LOV1 and LOV2) domains usually sense blue light (**Figures 2C, H**) (Suetsugu and Wada, 2012). In *Arabidopsis* blue light–activated PHOT1 (Kharshiing and Sinha, 2015) and PHOT2 have very similar properties (Jarillo et al., 1998; Christie and Briggs, 2001; Briggs and Christie, 2002; Staiger, 2008; Kharshiing and Sinha, 2015; Zhao et al., 2018b). Recently, the generation of a slow-photocycling variants of *phot1* or *phot2* was found to increase biomass production under light-limiting conditions due to improved sensitivity to light (Hart et al., 2019).

Zeitlupes (ZTL, FKF1, and LKP2) absorb blue light/UV-A (315–500 nm) and contain both photoreceptor and F-box protein activities within the same protein. Land plants encompass an extra family of UV-A/blue light photoreceptors that consist of ZTL, flavin-binding, kelch repeat, F-box (FKF1), and lov kelch protein2 (LKP2) proteins that are collectively named as Zeitlupes (Ito et al., 2012; Suetsugu and Wada, 2012). ZTLs form signaling complexes with factors of the circadian clock and photoperiodic flowering (Zoltowski and Imaizumi, 2014). ZTLs have a single FMN-binding LOV domain followed by an F-box and six-Kelch-repeat domain (**Figures 2D, H**) (Ito et al., 2012).

UV resistance locus 8 (UVR8) photoreceptor operates through UV-B light (280–315 nm) for UV acclimation in sunlight (Kaiserli and Jenkins, 2007; Rizzini et al., 2011; Rizzini et al., 2011; Christie et al., 2012; Christie et al., 2012; Jenkins, 2014b; Qian et al., 2016; Yin et al., 2016). UVR8 is a UV-B light photoreceptor using tryptophan residues as a chromophore (**Figure 2E, I**) to mediate phototropic bending, stomatal movement, and entrainment of the circadian clock (Fehér et al., 2011; Jenkins, 2014a; Tossi et al., 2014; Vandenbussche et al., 2014). UVR8 triggers large changes in gene expression leading to morphological adaptations and the production of flavonols to avoid the harmful effects of UV-B light (Favory et al., 2009; Rizzini et al., 2011; Jenkins, 2014b). Tomato SlUVR8 mediates plant acclimation to UV-B stress by orchestrating expression of the UVB-responsive genes such as elongated hypocotyl5 (*HY5*) and chalcone synthase (CHS) and accumulating UV-absorptive compounds. Furthermore, SlUVR8 enhances fruit chloroplast development accumulating transcription factor golden2-like 2 (SlGLK2) (Li et al., 2018).

### Interaction and Evolution of Photoreceptors and Membrane Transporters

Comprehensive knowledge on plant membrane transporters and photoreceptors systems along with the increasing number of sequenced plant genomes have enabled the comparative genomic and evolutionary analysis of many gene families (Cai et al., 2017; Chen et al., 2017; Wang et al., 2018; Zhao et al., 2019). Discovering orthologous membrane transporters and photoreceptors and deciphering their key functions have also led to a growing interest in the manipulation of these genes to enhance crop yield and quality (Boccalandro et al., 2003; Ganesan et al., 2017).

#### Evolution of Photoreceptors and Membrane Transporters

Photosynthetic organisms use a cluster of photoreceptors to sense the quality, quantity, and direction of light for photosynthesis and consequently growth and development over the long evolutionarily trajectory. Based on the evolutionary analysis of photoreceptors (Lariguet and Dunand, 2005; Zhao et al., 2019) and key membrane transporters (Cai et al., 2017; Chen et al., 2017; Wang et al., 2018; Li et al., 2019) across different land-plant and algal species, we proposed that evolutionary links between these photoreceptors and membrane transporters in various land-plant and algal species may provide clues toward light-induced interaction and potential co-evolution of photoreceptors with membrane transporters.

Photoreceptors have evolved across photosynthetic eukaryotes in land-plant species. Increase in the number of photoreceptors and function during plant evolution are associated with fitness enhancement (Rensing et al., 2016). Plant PHYs, PHOTs, CRYs, UVR8, and ZTLs originated in an ancestor of streptophytes (Li et al., 2015a; Zhao et al., 2019), and these photoreceptors mediated optimization of photosynthesis and photoprotection across green plants (Li et al., 2015b; Demarsy et al., 2018; Zhao et al., 2019). Liverworts, hornworts, and *Selaginella* have a single PHY, whereas mosses, lycophytes, ferns, and seed plants evolved diverse PHY sub-families due to independent gene duplications (Li et al., 2015a). PHOTs have been independent duplicated in most major land-plant lineages including mosses, lycophytes, ferns, and seed plants but had only single-copy genes in liverworts and hornworts (Li et al., 2015b). Four PHYs, two PHOTs, and five CRYs have been identified in the fern species *Adiantum capillus-veneris* (Kagawa et al., 2004). In addition, red/far-red and a blue light are sensed by a neochrome, a chimeric photoreceptor kinase combining a PHY photosensitive domain and PHOT, for phototropic response and chloroplast movement in *Adiantum capillus-veneris* (Nozue et al., 1998; Kawai et al., 2003). In mosses, liverworts, and ferns, chloroplast movement is enhanced by the PHYs, PHOTs, and/or neochromes in both red and blue lights, whereas rotation of the single chloroplast exhibits light-modulated chloroplast avoidance during the exposure of high light in green algae *Mougeotia* or *Mesotaenium* (Suetsugu et al., 2017). The liverwort *Marchantia polymorphia* shows early evolution of ZTLs in the plant lineage in the photoperiodic phase transition (Kubota et al., 2014). Moreover, the high levels of conserved structure of ZTL homologs among monocots and dicots suggest a certain similarity function of these genes across species (Taylor et al., 2010). UVR8 was evolutionarily well conserved within the green lineage, from green algae to angiosperms, and was expressed throughout the plant life cycle (Rizzini et al., 2011; Fernández et al., 2016; Tilbrook et al., 2016; Soriano et al., 2018; Zhao et al., 2019). For instance, in *Arabidopsis* and *Chlamydomonas reinhardtii*, only some families of photoreceptors are overlapped and preserved (Demarsy et al., 2018). *Arabidopsis* contains PHYs (*PHYA-E*), CRYs (*CRY1-2*), PHOTs (*PHOT1-2*), *ZTLs*, and UVR8 (Heijde and Ulm, 2012; Galvão and Fankhauser, 2015). By comparison, in the *C. reinhardtii*, UVR8 detects UV-B light whereas blue/UV-A is detected by one PHOT (PHOT1), two CRY-DASHs, one plant-like CRY (pCRY), and one animal-like CRY (aCRY) (Petroutsos, 2017). The latter shows responses both in blue and red lights (Beel et al., 2012; Kianianmomeni and Hallmann, 2014; Tilbrook et al., 2016). However, PHYs and ZTLs are not found in most of the algal species tested (Zhao et al., 2019). Instead, aureochrome (aureo) and opsin are identified in algae by recent genomic studies apart from major photoreceptors (Kianianmomeni and Hallmann, 2014; Essen et al., 2017; Kroth et al., 2017). The functional motifs of UVR8 are widely conserved in green algae, bryophytes, lycophytes, and angiosperms (Fernández et al., 2016), but not in gymnosperms because of redundancy of this photoreceptor or there is an alternative in gymnosperms (Tossi et al., 2019).

Plant membrane transporters have been co-evolved toward higher clades of plant throughout their evolutionary trajectory. The retention of these key membrane transporters and proteins could have had a profound effect on the adaptation of land species during evolution (Chen et al., 2017; Wang et al., 2018). Furthermore, most of the tested algae appeared not to have some families of the membrane transporters compared to plant species. For instance, *Arabidopsis* plasma membrane H^+^-ATPase (AHAs) autoinhibited Ca^2+^-ATPase (ACAs); Na^+^/H^+^ antiporter (NHXs), ABCs, CLCs, and CAXs are found amongst plants and algae during evolution, whereas SLACs, ALMTs, and high-affinity K^+^/Na^+^ transporter (HKTs) seem to be evolved after the colonization of terrestrial habitats by land plants (Cai et al., 2017; Chen et al., 2017; Wang et al., 2018). The high conservation of transporter protein families points to early acquisition of active control membrane transport mechanisms in plants (Chater et al., 2011; Ruszala et al., 2011; Komatsu et al., 2013; Lind et al., 2015; Chen et al., 2017). Interestingly, homologs of many *Arabidopsis* transporters such as SLAC1, ALMT12, TPK1, slow vacuolar Ca^2+^ channel (TPC1), and AHA1 have been found in all land-plant species studied (Cai et al., 2017; Cao, 2019), which are consistent with the evolution of photoreceptors (Zhao et al., 2019). Moreover, comparative genomic analysis revealed the evolutionary conservation of aquaporins, such as the plasma membrane intrinsic proteins (PIPs) family from algae to angiosperm in the long-term natural selection of land plants (Cai et al., 2017; Chen et al., 2017; Wang et al., 2018; Li et al., 2019). In the interaction between photoreceptors and membrane transporters, light-dependent production of assimilates regulates AKT2/3 transcript through a CO_2_-dependent mechanism in *Arabidopsis* (Deeken et al., 2000). The moss *P. patens* displays light-induced action potential changes and its chloroplasts, and PHYs are essential for plasma membrane channels’ activation through changes in cytosolic Ca^2+^ (Ermolayeva et al., 1996; Koselski et al., 2008). Light-dependent changes in plasma membrane ion transport have also been studied in the algae *Eremosphaera viridis* (Schönknecht et al., 1998) and *C. reinhardtii* (Nagel et al., 2002; Nagel et al., 2003), where light regulates phototactic and photophobic responses with the initiation of channelrhodopsins 1 and 2 (ChR1 and ChR2). The currents conducted by channelrhodopsins in high light intensities are likely to depolarize the *Chlamydomonas* plasma membrane with the mediation mostly of proton/cation flow (Harz and Hegemann, 1991; Harz et al., 1992; Schneider et al., 2015). Are these membrane transporters co-evolved with the photoreceptors during the evolutionary of plants from aquatic to terrestrial life? Is there any direct or indirect regulation of these photoreceptors on the membrane transporters in order to transduce the light signals?

Exploration of the evolution of membrane transporters and photoreceptors can be advantageous with these evolutionary genomics and bioinformatics techniques toward better understanding of their molecular evolutions throughout the evolutionary trajectory. Nevertheless, further research is needed to answer these questions combining the evolutionary analysis with their functional domains, cloning the key genes, and complementing or overexpressing these genes in different plant species.

#### Regulation of Photoreceptors on Plasma Membrane Transport

Here, we summarize the identified photoreceptors and their interactions with the down-stream signaling components in plants. PHOTs are the obvious candidates for this role as these protein kinases are associated with the plasma membrane (Briggs and Christie, 2002; Sakamoto and Briggs, 2002). CRYs predominantly localized within the nucleus (Wu and Spalding, 2007) are unlikely to directly regulate membrane transport. PHYs are able to affect membrane transport, at least in mosses (Ermolayeva et al., 1996; Ermolayeva et al., 1997). Furthermore, ZTLs mainly regulate circadian rhythms and are unlikely to have a strong influence on membrane transport (Más et al., 2003; Demarsy and Fankhauser, 2009).

PHY-induced inward Ca^2+^ current immediately starts to depolarize the membrane while concurrently increasing [Ca^2+^]_cyt_ (Ermolayeva et al., 1996; Ermolayeva et al., 1997). Irradiation of dark-adapted caulonemal filaments of *P. patens* with red light evoked changes of E_m_. The transient depolarization was blocked by application of far-red light, K^+^ channel–blocker tetraethylammonium, the Cl^−^ channel–blocker niflumic acid, removing Ca^2+^ from the external medium or replacing Ca^2+^ with Mg^2+^. Thus, K^+^, Cl^−^, and Ca^2+^ transporters may be involved in the early events in the PHY signaling pathway (Ermolayeva et al., 1996). Moreover, voltage clamp and ion flux measurements showed that K^+^ and Ca^2+^ channels are activated at the red light–induced depolarization, indicating red light–induced PHYs and ion channel interaction at the plasma membrane (Ermolayeva et al., 1997). However, research on direct PHY-mediated effects on membrane transporters are yet to be demonstrated.

PHOT1 and PHOT2 regulate membrane transport *via* the changes of [Ca^2+^]_cyt_ in plants (Harada et al., 2003). It was suggested that PHOT1 regulates blue light–induced Ca^2+^ uptake into the cytoplasm from the apoplast in etiolated *Arabidopsis* seedlings and significant changes in Ca^2+^ and H^+^ fluxes (Babourina et al., 2002). It was shown that blue light activates voltage-dependent calcium-permeable channels in the plasma membrane of mesophyll cells. Blue-light stimulated photoreceptors control of Ca^2+^-channel activity, which was dramatically reduced in *phot1-5* and was eliminated in the double mutant *phot1-5phot2-1*. By contrast, in *cry1-3cry2-1* double mutant, the Ca^2+^ channel remained sensitive to blue light (Stoelzle et al., 2003). PHOTs can also affect ion transport in the long-term through modulation of auxin transport on *Zea mays* K^+^ channel 1 (ZMK1) (Fuchs et al., 2003). Although PHOT signaling has been intensively studied, there are still unresolved nodes for the blue light signal to be passed onto membrane transport proteins.

CRYs exported to the cytoplasm upon illumination (Yang et al., 2000) can potentially interact with plasma membrane transporter proteins. Mutant *hy4* lacking the *CRY1* exhibits a membrane depolarization merely 30% of the wild-type magnitude (Spalding, 2000), which indicates that CRY1 may indirectly modify ionic currents across the plasma membrane (Parks et al., 1998). The excitation of CRY1 by blue light leads to the activation of anion channels at the plasma membrane of etiolated *Arabidopsis* hypocotyl cells within seconds (Cho and Spalding, 1996), resulting in membrane depolarization (Parks et al., 1998). The rapidity of its action may be explained by the presence of significant fractions of functional CRY1 in the cytoplasm of *Arabidopsis* hypocotyl cells or the emerging of CRY1-dependent signal from the nucleus to regulate channel gating within the first few seconds of illumination (Guo et al., 1999). However, this link is still largely unresolved due to limited advancement in this area of research.

#### Photoreceptors and Membrane Transporters in Light Signaling Network

**Figure 3:**
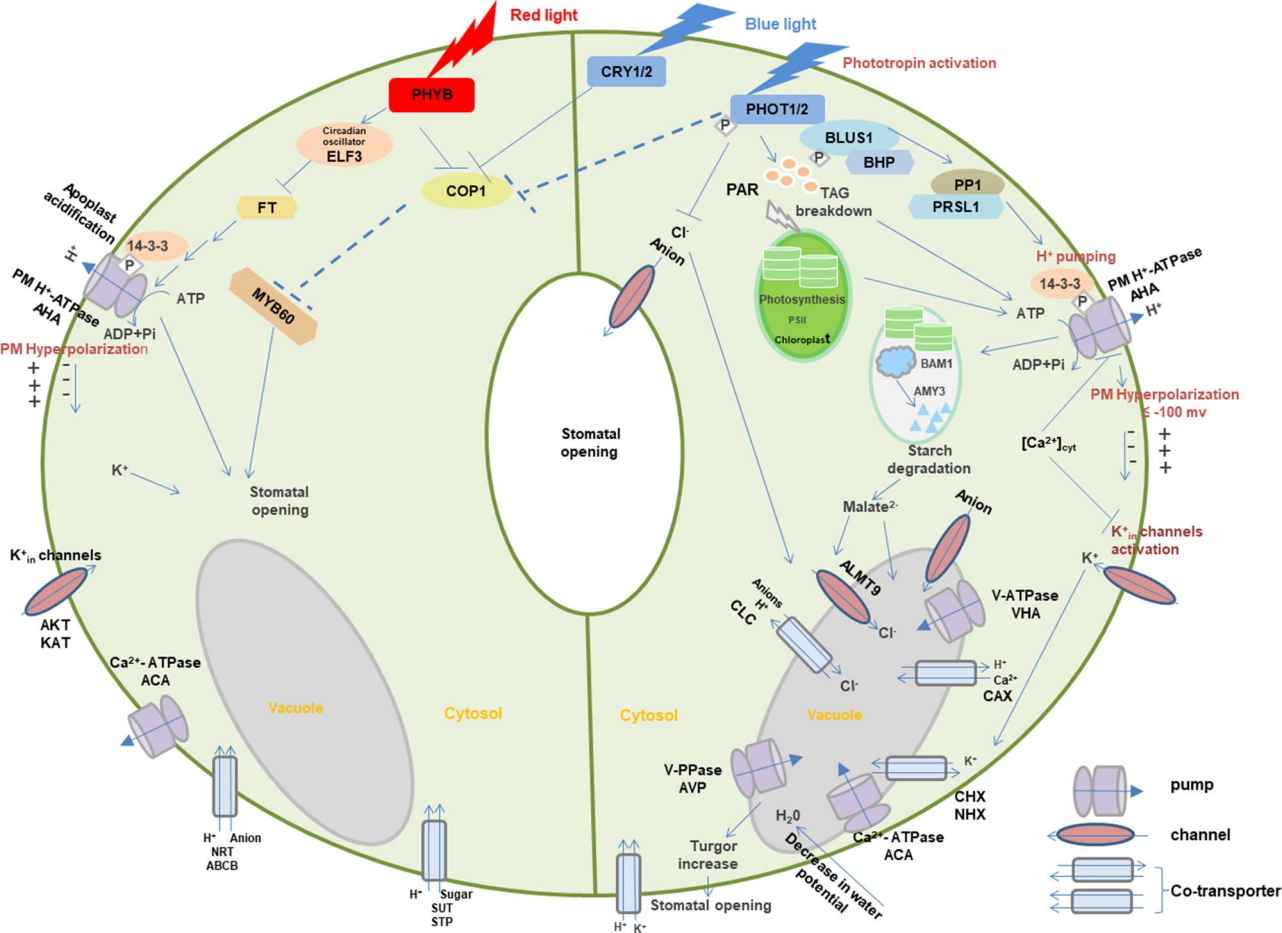
Light-induced interaction between photoreceptors and membrane transporters in stomatal guard cells. In blue light, PHOTs are autophosphorylated and start a signaling cascade to activate H^+^-ATPase which eventually results in the opening of stomata through the signals transduced downstream with the generation of different substrates such as BLUS1, PP1, and 14-3-3 protein. Blue light–induced H^+^-ATPase activation causes the hyperpolarization of the plasma membrane followed by the activation of inward-rectifying K^+^ channels to accumulate K^+^ ions, resulting in the turgor increase that leads to stomatal opening. Moreover, blue light-mediated signaling downstream of H^+^-ATPase activity degrades starch in guard cell chloroplasts. Blue light–induced stomatal regulation is also mediated by CRYs. Red light regulates stomatal opening through signaling transduction of PHYB, COP1, ELF3, FT,14-3-3 protein, AHA, KAT, ACA, SUT, NRT, and potentially MYBs. Arrows, T-bars, and dotted T-bar lines represent positive, negative, and hypothetical regulations, respectively. The P in the white rhombus indicates phosphorylation of proteins. PHOT, Phototropin; BLUS1, blue light signaling1; BHP, blue light-dependent H^+^-ATPase phosphorylation; PP1, type 1 protein phosphatase; PRSL1, regulatory subunit of protein phosphatase 1; 14-3-3, 14-3-3 protein; TAG, triacylglycerol; CRY, cryptochrome; PHY, phytochrome; COP1, constitutive photomorphogenic1; ELF3, early flowering 3; FT, flowering locus t; ALMT, aluminum-activated malate transporter; CLC, anion channel/anion/H^+^ antiporter; PAR, photosynthetically active radiation; PM, plasma membrane. AHA, plasma membrane H^+^-ATPase; VHA, vacuolar H^+^-ATPase; ACA, Ca^2+^-ATPase; AVP, vacuolar H^+^/K^+^-PPase; AKT, KAT, and KC, K^+^ inward-rectifying channels; CAX, Ca^2+^/H^+^ antiporter; CHX, cation/H^+^ exchanger; NHX, Na^+^(K^+^)/H^+^ antiporter; SUT, sucrose transporter; STP, monosaccharide/H^+^ symporter; NRT, nitrate transporter; ABCB, ATP-binding Cassette transporter. Models are adapted from (Shimazaki et al., 2007; Chen and Blatt, 2010; Chen et al., 2012a; Inoue and Kinoshita, 2017; Ando and Kinoshita, 2018).

Photoreceptors may coordinate with membrane transporters in light-induced signaling transduction. Here, guard cells are used to demonstrate the role of light-dependent photoreceptors and membrane transport in stomatal opening (**Figure 3**) (Roelfsema and Hedrich, 2005; Chen et al., 2012a; Inoue and Kinoshita, 2017; Ando and Kinoshita, 2018).

When guard cells are illuminated with blue light, PHOTs are triggered through auto-phosphorylation for the initiation of signaling for stomatal opening (Kinoshita et al., 2001; Christie, 2007). Upon blue light, two Ser residues are auto-phosphorylated in the kinase activation loop of PHOTs, and phosphorylation is essential to transduce signaling downstream through substrate recognition (Inoue et al., 2008; Inoue et al., 2010; Inoue et al., 2011). The activated PHOTs directly phosphorylate another protein kinase blue light signaling1 (BLUS1), which indirectly conveys the signal to type 1 protein phosphatase (PP1) and its regulatory subunit PRSL1 (Takemiya et al., 2006; Takemiya et al., 2012; Takemiya et al., 2013; Takemiya and Shimazaki, 2016). Furthermore, a Raf-like protein kinase, blue light–dependent H^+^-ATPase phosphorylation (BHP) bound to BLUS1 forms an early signaling complex with PHOTs to facilitate phosphorylation of a second last Thr of the PM H^+^-ATPase (Hayashi et al., 2017). The signal produced by BLUS1 finally triggers the PM H^+^-ATPase in guard cells through phosphorylation of Thr in the C terminus with successive binding of the 14-3-3 protein (Shimazaki et al., 2007; Hayashi et al., 2011; Yamauchi et al., 2016). H^+^-ATPase activation in blue light drives H^+^ pumping and causes the hyperpolarization of the plasma membrane (Shimazaki et al., 2007; Marten et al., 2010). Besides, red light–induced fluency rate-dependent PM H^+^-ATPase phosphorylation in guard cells (Ando and Kinoshita, 2018) promotes stomatal opening (Ando and Kinoshita, 2019). This hyperpolarization activates inward-rectifying K^+^ (K^+^_in_) channels (Lebaudy et al., 2008; Kim et al., 2010), which leads to the accumulation of K^+^. Water potential of guard cells is decreased due to accumulation of ions, resulting in water uptake into the vacuole accompanied with the turgor increase for stomatal opening (Inoue et al., 2010; Marten et al., 2010). Moreover, PHOT-mediated signaling downstream of H^+^-ATPase activity degrades starch in guard cell chloroplasts (Santelia and Lunn, 2017), which thus leads to stomatal opening possibly through malate synthesis involving β-amylase 1 (BAM1) and α-amylase 3 (AMY3) (Shimazaki et al., 2007; Horrer et al., 2016).

Blue light–induced stomatal regulation is also mediated by CRYs, interacting with PHYB-regulated red light–induced stomatal movement. COP1 plays a negative role in CRYs- and PHYB-induced signaling pathways (Chen et al., 2012a). Furthermore, stomatal aperture may be regulated downstream of the photoreceptors by the transcription factor such as MYB60 (Chen et al., 2012a). Circadian clock protein early flowering 3 (ELF3) and flowering locus t (FT) are also the important intermediates in the red light signaling pathway for stomatal regulation (Hicks et al., 2001). These signals are likely to regulate photoreceptor-mediated stomatal opening in different light *via* the modulation of ion transport through various channels and co-transporters with the light activation of pumps. However, many signaling components are still missing in the light-induced stomatal regulation, which requires extensive investigation in the future.

### Conclusions and Future Perspective

Although a great deal of research has been undertaken to study the effects of light irradiances on the plant growth and development, very little is known about the light-induced mechanisms involved in ion transport regulation of plants. An emphasis on the light-regulated membrane transport processes is necessary to synchronize light responses with receptor recognition, cellular homeostasis, and developmental programming. Establishing crucial links between ion transporters and photoreceptors recognition in light signaling transduction are only beginning to be explored. A large number of genes encoding photoreceptors and their potential downstream interacting transporters are identified in light-induced signaling pathway, but how these proteins interact to achieve rapid signaling transductions are yet to be investigated. Moreover, the knowledge of photoreceptors and downstream signaling pathways is, to a large extent, based on extensive studies in *Arabidopsis*. Few studies have focused on regulation of membrane transport and their interactions with photoreceptors in other species, especially in crops and early land plants. Thus, a deeper understanding of the molecular evolution and interaction of plasma membrane ion transporters with photoreceptors can shed new light on plant ecological conservation as well as agricultural production and crop quality, bringing potential nutrition and health benefits to animals and humans.

## Author Contributions

Z-HC and MB conceived and designed the research. SC and GC conducted the literature search and protein domain analysis. MB and Z-HC wrote the manuscript with contributions from DT and CC.

## Conflict of Interest

The authors declare that the research was conducted in the absence of any commercial or financial relationships that could be construed as a potential conflict of interest.

The handling editor is currently organizing a Research Topic with one of the authors, Z-HC, and confirms the absence of any other collaboration.
